# C57BL/6J Mice Are Not Suitable for Modeling Severe SARS-CoV-2 Beta and Gamma Variant Infection

**DOI:** 10.3390/v14050966

**Published:** 2022-05-05

**Authors:** Joshua M. Currey, Felix Rabito, Nicholas J. Maness, Robert V. Blair, Jay Rappaport, Xuebin Qin, Jay K. Kolls, Akhilesh K. Srivastava

**Affiliations:** 1 Tulane National Primate Research Center, Covington, LA 70433, USA; jcurrey@tulane.edu (J.M.C.); nmaness@tulane.edu (N.J.M.); rblair3@tulane.edu (R.V.B.); jrapport@tulane.edu (J.R.); xqin2@tulane.edu (X.Q.); 2 Department of Microbiology and Immunology, Tulane University School of Medicine, New Orleans, LA 70112, USA; 3 Center for Translational Research in Infection and Inflammation, John W. Deming Department of Medicine, Tulane University School of Medicine, New Orleans, LA 70112, USA; frabito@tulane.edu (F.R.); asrivastava1@tulane.edu (A.K.S.); 4 Department of Pediatrics, Tulane University School of Medicine, New Orleans, LA 70112, USA

**Keywords:** SARS-CoV-2, variants of concern, immunocompromised mice

## Abstract

SARS-CoV-2 variants, including B.1.1.7 (Alpha), B.1.351 (Beta), P.1 (Gamma), and B.1.617.2 (Delta) variants, have displayed increased transmissibility and, therefore, have been categorized as variants of concern (VOCs). The pervasiveness of VOCs suggests a high probability of future mutations that may lead to increased virulence. Prior reports have shown that VOC infection without expression of human angiotensin converting enzyme-2 receptor (hACE2) in mice is possible. We sought to understand if the increased transmissibility of VOCs can infect C57BL/6 mice without expression of hACE2 receptor required for entry of SARS-CoV-2 normally. We examined the ability of infection with Beta and Gamma variants to infect and cause both pathological and clinical changes consistent with severe COVID-19, including body weight changes, survival, subgenomic viral titer, lung histology on Hematoxylin and Eosin (H&E) staining, and viral protein expression as measured by immunohistochemistry staining of viral antigen (IHC). These methods were used to examine three groups of mice: C57BL6, *Rag2*-/-, and *Ccr2*-/- mice. We observed that these mice, infected with Beta and Gamma variants of SARS-CoV-2, did not show pathological changes as indicated by weight loss, altered survival, or significant lung pathology on H&E staining. Subgenomic qPCR and IHC staining for viral protein indicated that there was some evidence of infection but far below ACE2 transgenic mice, which showed clinical disease and pathologic changes consistent with ARDS. These data suggest that these variants replicate poorly even in the setting of profound immune deficiency.

## 1. Introduction

SARS-CoV-2 continues to circulate and mutate rapidly with several different variants, including B.1.1.7 (Alpha), B.1.351 (Beta), P.1 (Gamma), and B.1.617.2 (Delta) variants. The appearance of new variants, including Beta and Gamma, may decrease the effectiveness of the currently available vaccines. These variants have also displayed increased transmissibility and, therefore, have been categorized as variants of concern (VOCs) by the World Health Organization (WHO). The pervasiveness of these variants suggests a high probability of future mutations that may lead to increased virulence [1,2].

The Beta variant arose at the end of 2020 in South Africa, with multiple notable mutations in the spike. The N501Y mutation in spike has been implicated in facilitating enhanced binding to the humanized angiotensin converting enzyme 2 receptor (hACE2) than that seen in the Alpha and other lineages [3]. Two other notable mutations, E484K and K417N, have been shown to enhance immune evasion by escaping neutralizing antibody responses [1,2]. The Gamma variant, which was identified shortly after the Beta variant in December of 2020, quickly became the dominant strain in Brazil [3]. N501Y, K417N, and E484K are also seen in the Gamma variant and have been implicated along with the Beta variant in increasing the transmissibility of SARS-CoV-2 [1,2,3,4].

Notably, these VOCs have been reported to infect mice without hACE2 and replicate with relatively high viral titers within a few days post-inoculation [1,2,3,4]. Whether this is due to any of the notable mutations of interest seen in VOCs is unknown. Despite the ability to infect mice without hACE2 early post-inoculation, there are no indications of whether C57BL/6 mice can model severe COVID-19 with pathological changes consistent with ARDS [1,2,3,4]. To examine this, we used mice lacking either adaptive immunity (provided by T and B cells) or CCR2+ inflammatory monocytes to optimize the likelihood of observing severe pathology.

## 2. Materials and Methods

### 2.1. Mice and Ethics Statement

Male wild-type (WT) C57BL/6J (B6) mice were housed and bred in the animal facility of Tulane University School of Medicine. The mice (6 to 10 weeks old) were purchased from commercial sources and used in this study: (i) wild-type mice, (ii) *Rag2-/-* mice, which have a disruption of the recombination activating gene 2 (*Rag2*) and fail to generate mature T or B lymphocytes, (iii) *Rag2*/*IL2rg* mice, also known as *Rag2-/- γc-* mice, which exhibit T cell, B cell and NK cell immunodeficiency, and (iv) *Ccr2-/-* mice, which have absence of the CCR2 chemokine receptor, which affects monocyte/macrophage infiltration and makes them highly susceptible to pulmonary infection. The International Care and Use Committee of Tulane University reviewed and approved all procedures for this experiment (permit number P0443). The vivarial facilities at Tulane National Primate Research Center (TNPRC) are fully accredited by the Association for Assessment and Accreditation of Laboratory Animal Care (AAALAC).

### 2.2. In Vivo Infection to SARS-CoV-2 Variants

Wild-type B6 (*n* = 5), *Rag2-/-* (*n* = 5), *Rag2/IL2rg-/-* (*n* = 4), and *CCR2*-/- (*n* = 5) mice were infected intranasally with 2 × 105 TCID50 of Brazilian SARS-CoV-2 variant and wild-type (*n* = 5), *Rag2*-/- (*n* = 5), *Rag2/IL2rg*-/- (*n* = 4), and *CCR2*-/- (*n* = 5) mice were infected intranasally with 1.5 × 104 TCID50 of South African SARS-CoV-2 variant in the BSL3 facility; their survival and daily weight was recorded for 7 days post-infection, keeping criteria to euthanize the mice if there was greater than 20% weight loss.

### 2.3. Lung Tissue Processing

On day 7 post-infection, all surviving animals were euthanized, and the left lung was collected in TRIzol™ reagent (ThermoFisher) on ice for RNA isolation for subgenomic viral load. The right lung was collected in aqueous zinc formalin (Anatech) for histology and viral staining.

### 2.4. Real-Time PCR

RNA was isolated as per manufacture protocol for RNA extraction using TRIzol™ reagent. Lung RNA samples from wild-type (*n* = 5), *Rag2*-/- (*n* = 5), *Rag2*/*IL2rg*-/- (*n* = 4), and CCR2-/- (*n* = 5) mice infected with the Brazilian COVID-19 variant, and wild-type (*n* = 5), *Rag2*-/- (*n* = 5), *Rag2*/*IL2rg*-/- (*n* = 4), and CCR2-/- (*n* = 4) mice infected with the SA COVID-19 variant were taken for cDNA synthesis using iScript reverse transcriptase master mix (Bio-Rad Laboratories). Quantitative real-time PCR was performed on the Bio-Rad-CFX96 for subgenomic viral load determination using a published assay [5,6]. Viral loads were extrapolated from the COVID-19 subgenomic standard curve. A copy number of 1 was assigned to the samples which had no detection. The viral copy numbers from the lung are represented as copies/100 ng of RNA.

### 2.5. Lung Histology and Viral Staining

Tissues collected at necropsy were placed in formalin fixative for 30 days prior to being processed routinely [7]. Lung tissues were embedded in paraffin and cut into 5 um sections and either stained routinely or with immunohistochemistry. Stained tissue sections were scanned using a Zeiss Axio Scan.Z1 and whole slide images were viewed and captured using HALO image analysis software (HALO v3.2, Indica Labs, Albuquerque, NM, USA) [7].

Immunohistochemistry was performed on 5 um sections of FFPE tissue. Sections were baked overnight at 60 °C. They were then dewaxed and rehydrated with xylene, graded ethanol, and dd water as described in [2]. Slides were retrieved using a microwave oven heating method in Tris–EDTA buffer (ab93684, Abcam) for 20 min and cooled to room temperature. Sections were incubated with endogenous blocking solution (SP-6000-100, vector lab) for 10 min and 2.5% normal goat serum for 20 min at room temperature. Primary antibodies were incubated overnight at 4 °C, and then the slides were incubated with HRP horse anti-Rabbit IgG reagent for 30 min at room temperature. Immunoreactivity was detected by developing with DAB chromogen for 60 min.

## 3. Results

Here, we used both *Rag2*-/- and *Ccr2*-/- mice, on a C57BL/6 background, which exhibit a lack of matured T and B cells [8] or have a deficiency in monocyte recruitment during immune responses, respectively [9]. *Rag2*-/- mice are deficient in the *Rag2* gene, which is critical for (V(D)J) recombination during the process of B and T cell maturation [8]. *Ccr2*-/- mice are deficient in the CCR2 chemokine receptor, which is essential for the recruitment of monocyte/macrophages to sites of inflammation [9]. These mice were each challenged with the Beta and Gamma variants. The outcome of exposure was determined based on body weight changes, survival, lung histology, and measures of virus in lung tissue as determined by immunohistochemistry.

**Figure 1:**
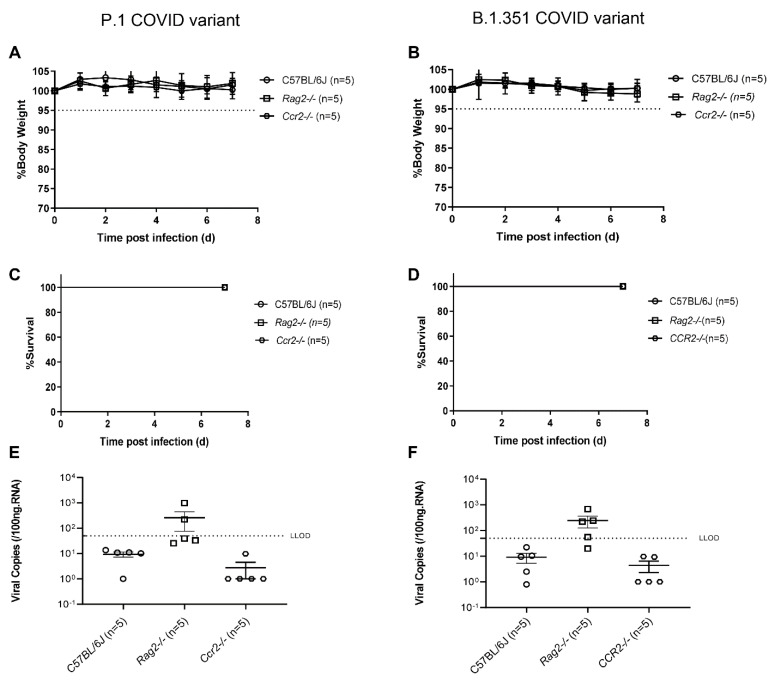
C57BL/6J (n = 5), *Rag2-/-* (n = 5), or *Ccr2-/-* (n = 5) mice were infected intranasally with 2 × 105 TCID50 of P.1 COVID variant (**A**,**C**,**E**) or with 1.5 × 10^4^ TCID50 of the B.1.351 COVID variant (**B**,**D**,**F**). Weight loss and survival was monitored and recorded for the next 7 days with criteria to euthanize the mice with a weight loss cutoff of 20% of their starting weight. On day 7, mice were euthanized and the SARS-CoV2 viral load (SgN copy number) was assayed by qPCR. None of the mice with either variant lost weight of more than 5% (**A**,**B**). Moreover, there was no mortality during the study period (**C**,**D**). Viral load was only reliably detected in the *Rag2-/-* mice (*p* < 0.05, compared to C57BL/6) but there were no statistical differences in viral loads using one way ANOVA and Tukey’s multiple comparisons test (**E**,**F**).

**Figure 2:**
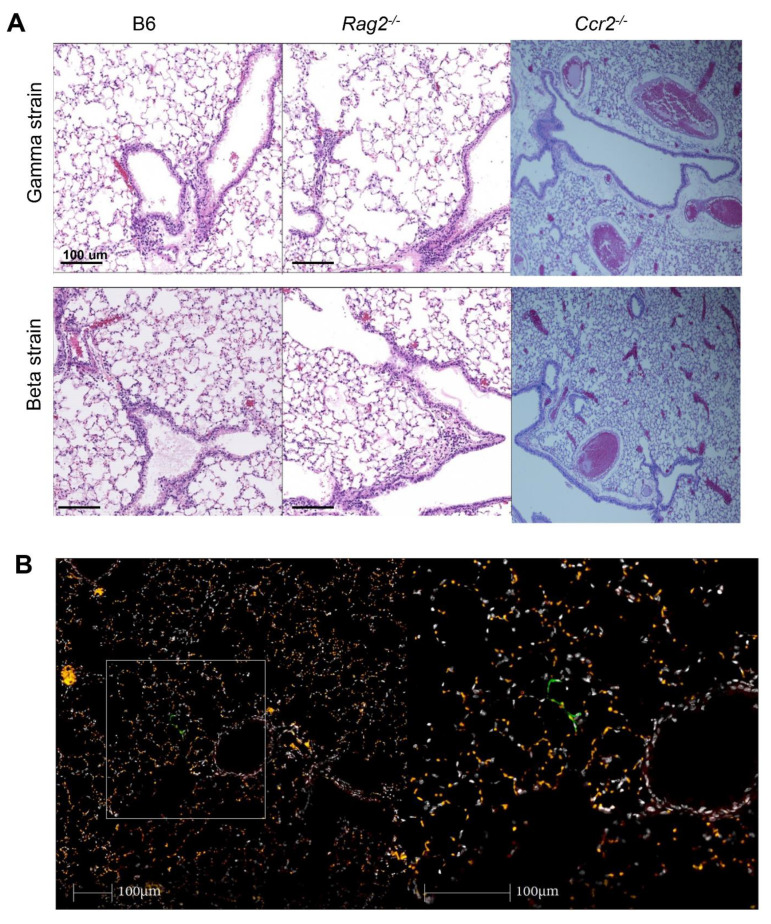
(**A**) Histological analysis of lungs of the mice infected with Gramma and Beta variants. Representative images shows the H&E staining in lungs of C57/B6 (B6) (n = 2), *Rag2^-/-^* (n = 2) and *Ccr2^-/-^* mice (n = 5). (**B**) Immunohistology staining of SARS-CoV-2 spike in *Rag2^-/-^* mice infected with Gamma variant. Green: SARS CoV-2 spike, Red: background staining, White; DAPI nuclear stain.

Previously, we demonstrated that K18-hACE2 mice inoculated intranasally with 1–2 × 10^5^ TCID50 showed a severe COVID-19 phenotype [4]. Based on our previous study, we used 1.5 × 10^5^ TCID50 of SARS-CoV-2 Beta or 2 × 10^5^ TCID50 of SARS-CoV-2 Gamma variants, to infect C57BL6 (*n* = 5), *Rag2*-/- (*n* = 5), and Ccr2-/- (*n* = 5) mice. We measured body weight daily post-exposure and found that there were no significant body weight changes among C57BL6, *Rag2*-/-, and *Ccr2*-/- mice infected with either Beta or Gamma variant (Figure 1A,B). No mice reached humane euthanasia endpoints (exhibiting >20% weight loss or abnormal behaviors) with either variant following exposure (Figure 1C,D). Subgenomic viral load was used to detect replicating virus in the lung of mice [4,6]. Both viral variants had significantly higher subgenomic viral loads in *Rag2*-/- compared to C57BL6 or *Ccr2*-/- mice (Figure 1E,F). At euthanasia, there were no significant histological changes in the lungs of the mice among the three groups (Figure 2A). We also detected SARS-CoV-2 spike antigen by immunohistochemistry in the lungs of *Rag2*-/- but not B6 or *Ccr2*-/- mice infected with the Gamma variant (Figure 2B). Together, these results indicate that neither wild-type or immunocompromised mice on a C57Bl/6 genetic background support high levels of SARS-CoV-2 variant infection and thus fail to serve as a model of severe COVID-19.

## 4. Discussion

Early studies suggested that these VOCs could replicate and cause some pathology in mice on a C57BL/6 genetic background. This observation was important as it would open the use of all the genetic tools available on the C57 background, and non-transgenic mice would have more natural expression of ACE2 when compared to hACE2 transgenic mice. C57 mice would, additionally, theoretically allow for the investigation of GWAS susceptibility loci using existing genetic tools on the C57BL6 background. Unfortunately, we found that viral infection was self-limiting even in the setting of profound immune deficiency. These data suggest that these VOCs are unable to support lung pathology and thus the combination of these VOCs and the various transgenic mice on the C57BL/6 background will have limited utility to investigate the genetic risk for severe COVID-19.

We attribute the lack of pathologic changes in *Rag2*-/- mice infected with VOCs to either a limited or absent inflammatory response. Our results differ slightly from Shuai et al., wherein they observed higher viral loads in C57BL/6 mice and some pulmonary pathology. However, this was at an earlier time point, 2 days post-exposure [1,3,4]. They did observe a significant reduction in viral load by day 7 post-exposure, similar to our findings reported here. Differences in experimental design may have accounted for the differences in our results. One difference is that they used ketamine for anesthesia, and we used a much briefer isoflurane anesthesia for inoculation. The longer effect of ketamine could influence the dwell time of the inoculum. Despite this, our data show a lack of lung pathology in mice lacking two critical pathways: in one strain lacking adaptive immunity and one strain unable to recruit monocyte-derived macrophages. Together, these results indicate that both murine strains are inadequate to model severe COVID-19 or COVID-19-related long term pulmonary disease with the current dose of virus. A higher dose of virus may be needed to infect both mouse strains to show pathological changes consistent with severe COVID-19.

In summary, our data support the need for further development of mouse-adapted strains on the C57Bl/6 background to further take advantage of the plethora of mouse genetic tools on the C57 background. Alternatively, hACE2 can be transduced in the lungs of those molecular-engineered mouse strains using various delivery systems [6]. Furthermore, previously, we and others have demonstrated that K18-hACE2 transgenic mice infected with SARS-CoV-2 show a severe COVID-19 phenotype [4,10,11,12]. Therefore, for pathogenesis studies, hACE2 introduction to genetically engineered mouse strains through a genetic cross with K18-ACE2 mice would theoretically sensitize those mice to severe SARS-CoV-2 infection [2,10,11,12].

## Data Availability

Data are contained within the article.
